# IL-1 blockade in inherited fever syndromes - what have we learnt?

**DOI:** 10.1186/1479-5876-9-S2-I6

**Published:** 2011-11-23

**Authors:** Helen J Lachmann

**Affiliations:** 1 UK National Amyloidosis Centre, Dept. of Medicine, University College London Medical School, London, UK

## 

The inherited periodic fever syndromes are single gene disorders of innate immunity which have provided invaluable insights into the regulation of inflammation. They are all extremely rare but are clinically important as they cause very significant day to day symptoms and carry a high risk of early death due to AA amyloidosis. With the exception of familial Mediterranean fever treatment was essentially ineffective until this century.

The discovery in 2002 that specific IL-1 blockade completely prevented all symptoms in cryopyrin associated periodic syndrome (CAPS) transformed treatment of this rare genetic disease. Isolation of the responsible gene, NLRP3, followed by rapid advances in molecular biology and identification of the inflammasome has made it clear that CAPS is purely a disease of IL-1 dysregulation. These discoveries combined with the availability of specific anti IL-1 agents have resulted in new insights into the regulation of IL-1 beta production in humans and its long term consequences.

IL-1 is now recognised to be an important mediator of inflammation in other inherited autoinflammatory diseases such as Familial Mediterranean fever (FMF), TNF receptor associated periodic syndrome (TRAPS), deficiency of IL-1 receptor antagonist (DIRA) and mevalonate kinase deficiency and has transformed patient prognosis and quality of life. In addition the recognition that IL-1 blockade provides complete disease control in acquired diseases of uncertain aetiology particularly Schnitzler’s syndrome and systemic onset juvenile chronic arthritis (SoJIA), adult onset Stills disease and recurrent idiopathic pericarditis has made it clear that these too are autoinflammatory disorders. One of the most striking features of these diseases is that IL-1 blocking agents have been used for prolonged periods as monotherapy without serious side effects or breakthrough of inflammatory activity thus differentiating them from the commoner autoimmune disorders such as rheumatoid arthritis and vasculitis.

